# Effect of pressure profile of shock waves on lipid membrane deformation

**DOI:** 10.1371/journal.pone.0212566

**Published:** 2019-02-21

**Authors:** Ralph Kfoury, Bahador Marzban, Emad Makki, Michael L. Greenfield, Hongyan Yuan

**Affiliations:** 1 Department of Mechanical, Industrial & Systems Engineering, University of Rhode Island, Kingston, RI, United States of America; 2 Department of Mechanical Engineering, College of Engineering and Islamic Architecture, Umm Al-Qura University, Makkah, Kingdom of Saudi Arabia; 3 Department of Chemical Engineering, University of Rhode Island, Kingston, RI, United States of America; University of Florida, UNITED STATES

## Abstract

Use of shock waves to temporarily increase the permeability of the cell membrane is a promising approach in drug delivery and gene therapy to allow the translocation of macromolecules and small polar molecules into the cytoplasm. Our understanding of how the characteristics of the pressure profile of shock waves, such as peak pressure and pulse duration, influences membrane properties is limited. Here we study the response of lipid bilayer membranes to shock pulses with different pressure profiles using atomistic molecular dynamics simulations. From our simulation results, we find that the transient deformation/disordering of the membrane depends on both the magnitude and the pulse duration of the pressure profile of the shock pulse. For a low pressure impulse, peak pressure has a dominant effect on membrane structural changes, while for the high pressure impulse, we find that there exists an optimal pulse duration at which membrane deformation/disordering is maximized.

## Introduction

Shock waves occur when a wave exceeds the local speed of sound in a fluid. They are characterized by discontinuous changes in pressure, temperature, and density of the medium in which they occur. In the case of shock wave impact on lipid bilayer membranes, the high particle velocity induced by the shock wave pulse can displace and disrupt the lipid molecules and increase the membrane permeability temporarily. Application of shock waves has been proven to increase permeability of the lipid bilayer allowing for delivery of drugs or oligonucleotides into the cell[1–3]. In addition to the direct impact of high speed shock waves on lipid membranes, it has been reported that shock waves induce microbubble collapse and formation of microjets as other mechanisms of the temporary change in membrane permeability [4], [5]. Both mechanisms involve the impact of high speed water molecules onto the membrane.

A shock wave is typically described by the pressure profile of the pulse, which can be characterized roughly by two characteristics: the peak pressure and the duration of the pulse. In an experimental study by Kodama et al.[2], by comparing different pressure profiles of shock waves, they found that a shock wave with a smaller peak pressure but longer pulse duration caused a greater increase in membrane permeability than a shock wave with higher peak pressure but shorter duration. They concluded that it was the pressure impulse (which is defined as the integral of the pressure over a time interval), rather than the peak pressure of the shock wave, that plays a dominant role in causing the membrane disruption.

Experimentally, it is difficult to image the membrane dynamics at the molecular scale due to the limitation of optical microscopy. Molecular dynamics (MD) simulations are well suited for studying membrane dynamics in the atomistic and molecular scales [6], [7]. Considerable research has been made to use MD simulations to ascertain the structural changes of lipid bilayer membranes subjected to shock waves and the effect of shock waves on membrane permeability [8–12]. Koshiyama et al.[13] modeled shock waves *in silico* on DPPC-containing lipid bilayer models. In their studies, changes in lipid membrane structure such as the collapse and rebound of the lipid bilayer, the increase in accumulated lateral displacement, and the temporal change of the instantaneous order parameter of the lipid bilayer due to shock waves were reported. Using a coarse-grained dissipative particle dynamics (DPD) model of the lipid bilayer, Ganzenmuller et al.[14] studied the critical velocity of shock wave impact beyond which the lipid membrane damage is not recoverable. In a recent study with molecular dynamics simulations, Zhang et al. studied the membrane response to uniaxial stretch with high loading rate [15]. Despite much progress in understanding membrane response to shock waves, knowledge of how membrane disruption depends on the pressure profile of shock waves (i.e., peak pressure, the duration of the pulse, and the pressure impulse) is still limited.

In this work, we use atomistic MD simulations to study the effect of the pressure impulse and the pulse duration (which can be considered as the reciprocal of the loading rate) of shock waves on membrane deformation/disordering. Simulations were carried out on lipid membrane models containing POPC (1-palmitoyl-2-oleoyl-sn-glycero-3-phoshocholine) with 10% cholesterol added. First, equilibrium simulations were run to measure membrane properties in the equilibrium state. Then, shock waves of varying pressure profiles were applied to the equilibrated system to ascertain the effect of varying shock characteristics such as loading rate on membrane structural properties.

## Methods

### Creation of lipid bilayer models

**Fig 1:**
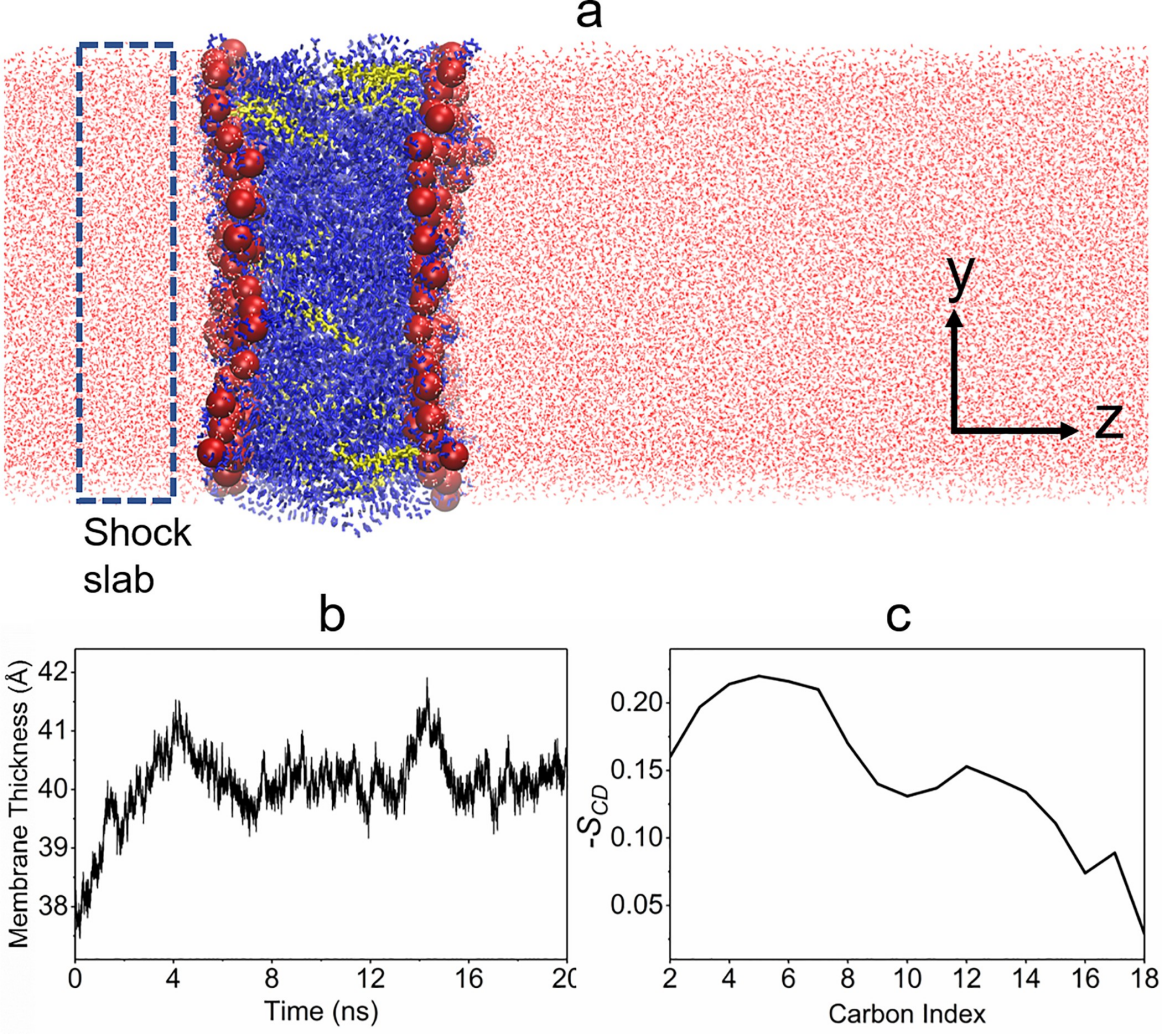
(a) Simulation snapshot of the equilibrated lipid membranes (POPC: blue, CHL: yellow, water: red, phosphorous atoms: red spheres), the rectangular box indicates the location of the water slab of thickness *L*_*w*_ for shock wave simulations. (b) The average bilayer thickness and (c) the order parameter (-S_CD_) of carbon-hydrogen bonds in acyl chains calculated over the course of 20 ns equilibration and sampling.

The membrane models tested in these simulations were created using the online software, CHARMM-GUI[16]. For the model tested in this research, POPC and cholesterol were chosen as the lipid species. The 10% cholesterol (9:1 POPC/CHL) membrane contained 216 POPC and 24 cholesterol molecules. Once the initial bilayer models were created and the membrane normal oriented in the z plane, they were solvated explicitly using the TIP3P[17] model for water molecules. Because in this work we only consider the mechanical behavior of the membrane, non-dissociating TIP3P water model is used in the shock wave simulations for its efficiency. A total of 230 Ångstroms (Å) of solvent was added in the z dimension. More specifically, 200 Å of solvent was added to the right of the bilayer model (z+) and 30 Å to the left (z-) (see Fig 1A). The values were chosen to ensure that the shockwave has sufficient distance to propagate in the z+ direction and enough solvent on left side of the membrane to apply a shockwave of varying pulse duration. Sodium and chlorine ions were added to the system until a concentration of 0.15M was reached. System composition and average sizes are listed in Table 1. The solvation and ionization was performed using the program VMD[18] (Visual Molecular Dynamics).

**Table 1:** Membrane properties averaged over 10 ns of sampling for 9:1 POPC/CHL lipid bilayer.

**Lipid Bilayer Composition**	216 POPC 24 CHL
**Total number of atoms** **(Including water)**	241968
**Number of water, Na+, Cl-** **molecules**	210852 198 198
**Average Membrane Thickness (Å)**	40.24 ±0.39
**Average Area per Lipid (POPC +CHL) (Å**^**2**^**)**	60.27±0.69
**Average Area per POPC (Å**^**2**^**)**	63.93±1.5
**Mean Order Parameter (Bilayer)**	0.149
**Mean Order Parameter** **(Upper Monolayer)**	0.150
**Mean Order Parameter** **(Lower Monolayer)**	0.148

### Equilibration of membrane models

An initial 10,000 steps of minimization was carried out in NAMD[19]. Following minimization, equilibration of the membrane model was carried out in an NPT (fixed number of moles (N), fixed pressure (P), fixed temperature (T)) ensemble at 310 K and 1 atm using a time step of 2 fs. The three orthogonal dimensions of the periodic cell were allowed to fluctuate semi-isotropically. Periodic boundary conditions were employed in all three dimensions. All hydrogen-containing bonds were constrained using the SHAKE algorithm. Constant temperature was maintained using Langevin dynamics with a damping coefficient of 1/ps. Constant pressure was controlled using a Nosé-Hoover Langevin piston algorithm with a Langevin piston period of 50 fs and a Langevin piston decay of 25 fs. The CHARMM36[20] force field for lipids and cholesterol was used. The equilibrations were carried out for 10 ns while system properties such as average bilayer thickness stabilized. Then, the next 10 ns were used for sampling runs to calculate relevant membrane properties. The restart files from these simulations were used as the starting files for the shock wave simulations. After 10 ns of equilibration and 10 ns of sampling, snapshots of the lipid membrane system were taken and are shown in Fig 1A.

### Shock wave implementation

The previously established methods of Koshiyama et al.[13] were used to model the shock waves in the following simulations. The pressure impulse $\hat{I}$ can be related to the change of momentum as

$$\hat{I}=\frac{\Delta M}{A}$$ (1)

where *A* is the area of the simulation box in the x-y plane and *ΔM* is the change of momentum. The shock waves in these simulations were modeled as a change of momentum to water molecules contained in a slab of volume *L*_*w*_*A* adjacent to the lipid bilayer (see Fig 1A); where *L*_*w*_ represents the thickness of the water slab and *A* represents the lateral area of the water slab in the x and y plane. This water slab will be termed the “shock slab” from this point on. To implement this change in momentum, an average velocity was added to the z-component of the thermal velocity of water atoms in the shock slab. The average velocity added to water molecules in the shock slab, denoted by Δ*v*_*z*_, was calculated using

$$\Delta v_{z}=\frac{\hat{I}}{\rho L_{w}}$$ (2)

where *ρ* is the density (i.e., mass per unit volume) of the water. Due to added velocity Δ*v*_*z*_, the shockwave will propagate along the z+ axis in the systems tested. In our simulations, the nominal pulse duration of the shock waves, denoted by *t*_*p*_, is defined as the pulse length divided by the added velocity:

$$t_{p}=\frac{L_{w}}{\Delta v_{z}}$$ (3)

Note that the pulse duration defined here is an approximation and is based on the square wave assumption. In fact, as indicated later on by the simulation results (Fig 3C and 3D), the initial square pressure wave becomes triangular shape shortly after the shock wave starts to propagate. For simplicity, we use the passing time of the initial water slab through a fixed point, i.e., *t*_*p*_, as the nominal pulse duration for the comparison between different pulses.

To facilitate the discussion in the Results Section, we denote the nominal peak pressure with *P*_*n*_. It is defined through Eq (1) so the pressure impulse equals the momentum increase: $\hat{I}=P_{n}t_{p}=\rho L_{w}\Delta v_{z}=\rho {\Delta v_{z}}^{2}t_{p}$. The last substitution uses the pulse duration and yields

$$P_{n}=\rho {\Delta v_{z}}^{2}$$ (4)

Note that the nominal peak pressure is only an approximation of the order of the magnitude of the peak pressure of the shock waves. As implied in Eqs (2)–(4), the thicker the shock slab, the smaller the added velocity (Δ*v*_*z*_), the lower the peak pressure of the impulse, but the longer the duration of the shock. As pointed out by Koshiyama et al.[21], the velocity Δ*v*_*z*_ does not correspond to the propagation speed of the shock wave. The shock wave simulations were carried out in an NVE (fixed number of moles (N), fixed volume (V), fixed energy (E)) ensemble to simulate non-equilibrium dynamics. The duration of each simulation was determined using the velocity applied and the length of the simulation box such that the simulation ended when the pulse would travel one box length in the z dimension. Therefore, no reflective waves are present in the simulations.

In this work, two sets of shock wave simulations were carried out. The first set of simulations carried out sought to analyze the effect of the gap (distance between the lipid membrane and applied shock wave) on the change in lipid membrane thickness due to shock. Shock impulses of 10 mPa·s (note that “mPa·s” represents 10^−3^ N/m^2^·s) were applied to the lipid membrane while varying the gap distance (5,10,15,20 Å). The second set of simulations performed aimed to study the effect of the loading rate (*γ*) of the applied shock wave on the lipid membrane properties. For a single pulse of the shock wave, the loading rate is defined as the reciprocal of the pulse duration,

$$\gamma =\frac{1}{t_{p}}$$ (5)

Shock waves with pressure impulses of 1 mPa·s and 10 mPa·s (note that “mPa·s” represents 10^−3^ N/m^2^·s) were applied on the lipid bilayer membrane. For each pressure impulse tested, the shock slab thicknesses (*L*_*w*_) simulated were 5, 10, 15, 20 and 30 Å. Correspondingly, the pulse duration ranges from 0.25 ps to 9 ps (for 1 mPa·s pressure impulse) and 0.025 ps to 0.9 ps (for 10 mPa·s pressure impulse). The peak nominal pressure calculated with Eq (4) ranges from 0.11 GPa to 4 GPa (for 1 mPa·s pressure impulse) and 11 GPa to 400 GPa (for 10 mPa·s pressure impulse). All-atom MD simulations are computationally very expensive.

To make the MD simulations feasible, the pulse duration are much shorter than those imposed experimentally and the peak nominal pressures imposed in the simulation are higher than those in the experiments [2]. In this work, only the shock waves with a normal incident angle were studied. A previous research [21] has studied the in-plane or lateral membrane responses to the shock waves with oblique incident angles, and found that water penetration is insensitive to the incident angle.

### Analysis

Analysis of lipid membrane structural properties after equilibration and post-shock wave application was carried out using the MEMBPLUGIN[22] software. MEMBPLUGIN was used for calculating average membrane properties such as the membrane thickness, area per lipid, and carbon-hydrogen order parameter of acyl chains of phospholipids. The *Membrane Thickness* tool contained in this software calculates the average membrane thickness over a chosen trajectory by measuring the distance between the two density peaks of user-selected atoms (phosphorous) belonging to the head group of POPC. In addition, the percent decrease in the average membrane thickness was calculated. The *Deuterium Order Parameter S*_*CD*_ tool calculates the carbon-deuterium order parameter of acyl chains in phospholipids. This parameter is typically derived through NMR experiments and represents the orientational mobility of the carbon-hydrogen methylene bonds along the lipid tails of the phospholipid model. Eq 3 is used to measure the *S*_*CD*_ parameter of carbon-hydrogen bonds in phospholipid tails in MEMBPLUGIN[22] software

$$-S_{CD}=-\langle \frac{3{cos}^{2}\theta -1}{2}\rangle$$ (6)

where *θ* is the instantaneous angle between a C-H (carbon-hydrogen) bond and the membrane normal (z-plane), −*S*_*CD*_ is the negative of *S*_*CD*_ so that −*S*_*CD*_ increases when the system is more ordered, with a maximum of ½. The averaging is done over all phospholipids (POPC) in the system.

When analyzing the deformation of the bilayer membrane subjected to the shock wave impact, the compressive strain is used, which is defined as the membrane thickness decrease divided by the original membrane thickness (i.e., the percent decrease in membrane thickness). The greatest percent decrease in membrane thickness during shock wave passing through the membrane is defined as the maximal compressive strain (denoted by *ε*_*max*_) and used as an indicator of membrane deformation in the analysis of the simulation results.

## Results and discussion

### Equilibration analysis

Fig 1A shows a snapshot of the equilibrated membrane after 20 ns simulation time. In Fig 1B, the average bilayer thickness is plotted throughout the course of equilibration and sampling. The membrane thickness reaches a dynamic equilibrium after about 8 ns from the beginning, so the latter 10 ns simulation was used for extracting equilibrium membrane properties. The mean membrane thickness averaged over the last 10 ns of sampling was 40.24 ±0.39 Å for the 9:1 POPC/CHL membrane. The average area per POPC of the 9:1 POPC/CHL membrane was 63.93±1.5 Å^2^, which is less than the value of 68.3 ± 1.5 Å^2^ determined computationally[20] and experimentally[23] for POPC-only membranes. The presence of cholesterol in lipid bilayers has been experimentally shown to reduce the average area per lipid [24]. Fig 1C plots the order parameter in both acyl chains (palmitoyl + oleoyl), averaged over 10 ns of sampling. Overall, the lower-indexed C-H bonds located closer to the phospholipid head group are more ordered than the higher-indexed C-H bonds further down the acyl chain. We will use the order parameter (-S_CD_) as a measure of the fluidity or disordering of lipids in the shock wave simulations below.

### Shock wave simulations

**Fig 2:**
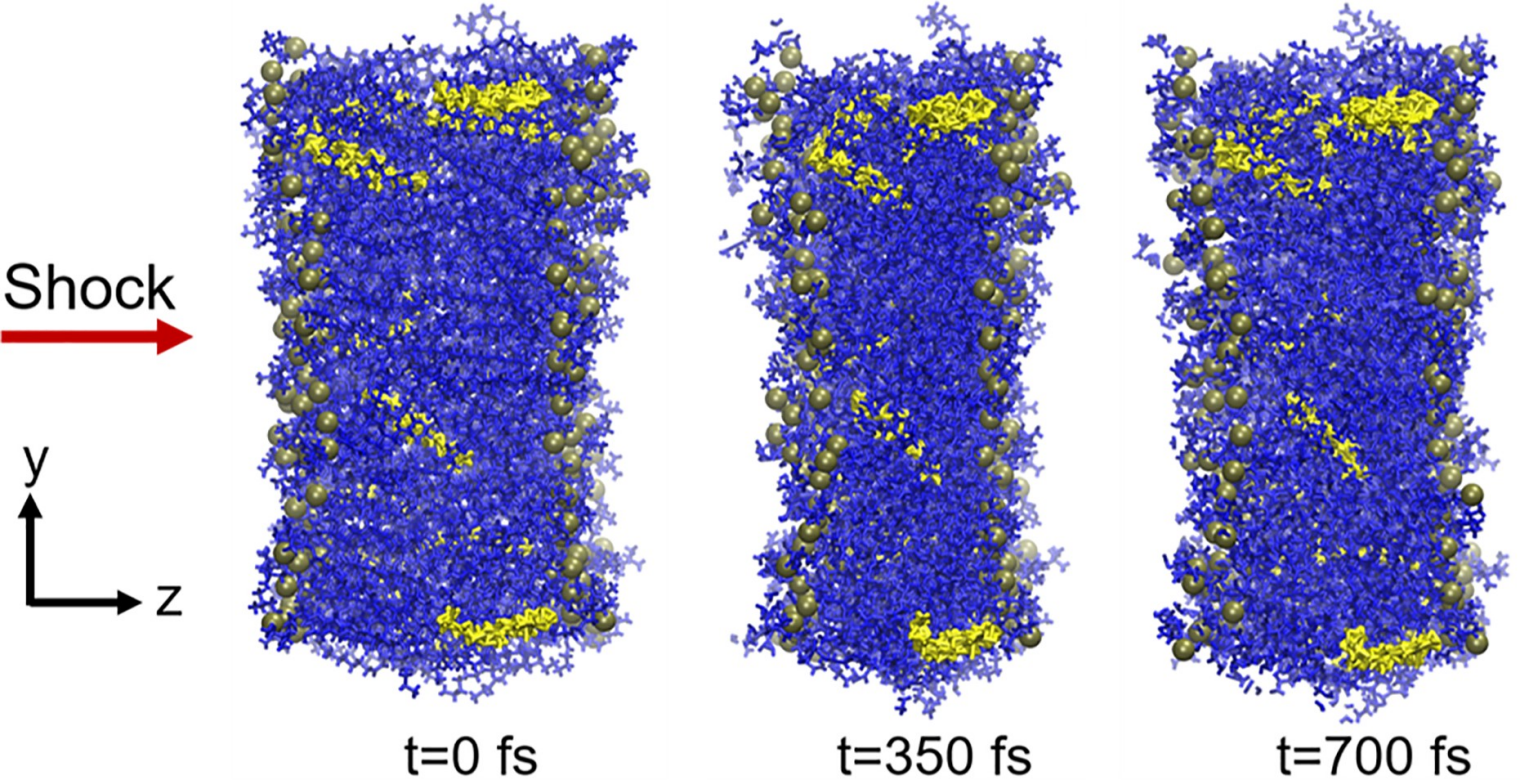
Simulation snapshots at 0 fs, 350 fs and 700 fs of the 9:1 POPC/CHL membrane model subjected to a 10 mPa·s pressure impulse.

The shock wave simulations were performed for a single pass of the shock wave through the membrane (see the details on simulation setup in Method section). As shown in Fig 2, the structural effect a shock impulse has on the lipid bilayer structure is a collapse of the bilayer leading to a reduction in the membrane thickness. Once the shock has propagated through the lipid bilayer, the membrane thickness starts to return to its original value. (See S1 Movie for a visual illustration of the response of the membrane to the shock wave impact.)

### Effect of the gap between the water slab and the membrane

**Fig 3:**
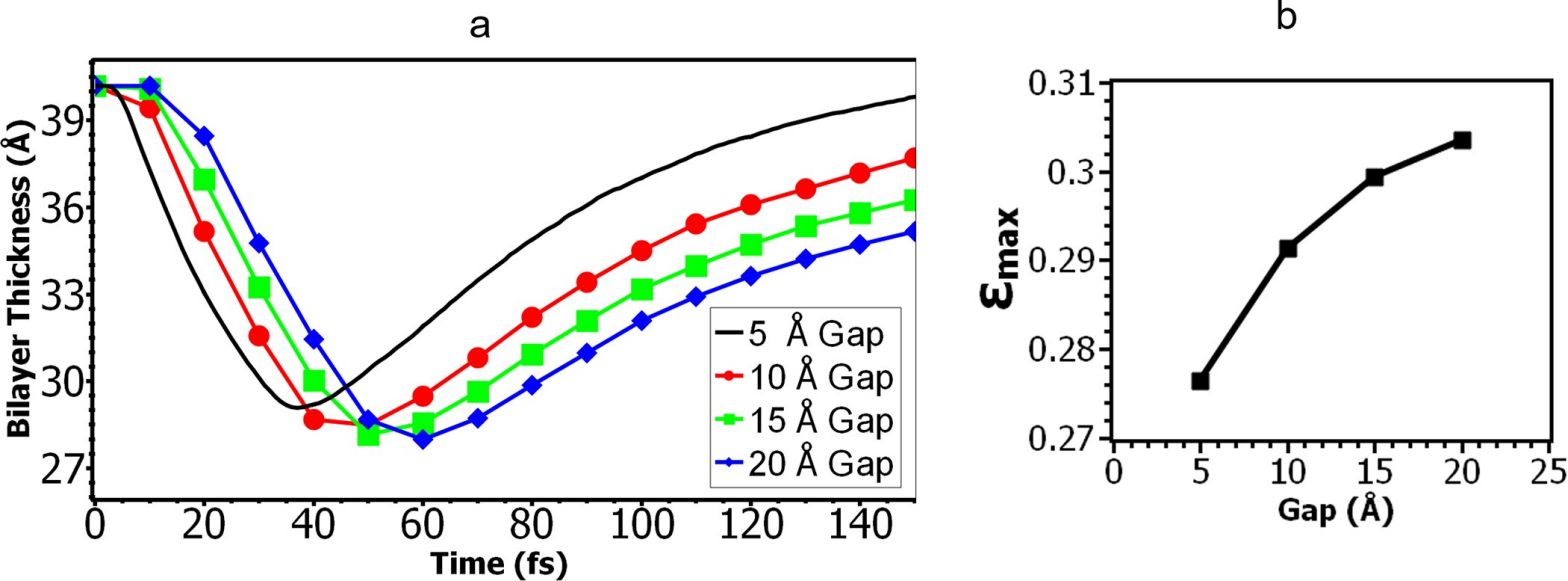
Effect of the gap between the water slab and the membrane on the change of membrane thickness upon the impact of shock waves. (a) Time history of the average membrane thickness of the lipid bilayer for different gap distances. (b) the maximal compressive strain as a function of the gap distance.

We first investigate the effect of the gap between the water slab and the membrane on the membrane dynamics upon the impact of shock waves. In this set of simulations, the same size of the water slab (i.e., the same length and same magnitude of added velocity) was used in the simulations; only the gap between the water slab and the membrane was varied. Time history of the average membrane thickness of the lipid bilayer for different gap distances is plotted in Fig 3A. The time at which the maximal membrane deformation occurs shifts due to the increased gap, as expected due to the shock slab being placed further from the lipid membrane.

Fig 3B shows that the magnitude of the maximal compressive strain (*ε*_*max*_) changes with the gap distance: as the gap becomes larger, the maximal membrane deformation increases. This is counterintuitive since the peak pressure hitting the membrane decreases when the gap is larger due to the decay of peak pressure over time. Our interpretation is that the membrane exhibits strain-thickening effect (e.g., cornstarch-water mixture) when subjected to large pressure impulses. As the gap is increased, the duration of the pressure pulse hitting the membrane becomes longer, the loading rate is smaller, so the membrane becomes “thinner” and easier to deform.

### Effect of the loading rate

We further study the effect of the loading rate *γ*, defined in Eq (5), on the membrane dynamics in the second set of simulations. The gap between the lipid membrane and shock slab was fixed. Having the same gap in these simulations allows us to exclude the influence of the gap on membrane properties in response to shock. While the gap was fixed, the pulse duration (thickness of shock slab) was varied to alter the loading rate of the shock wave. The equilibrated membrane was then subjected to pressure impulses of 1 and 10 mPa·s.

**Fig 4:**
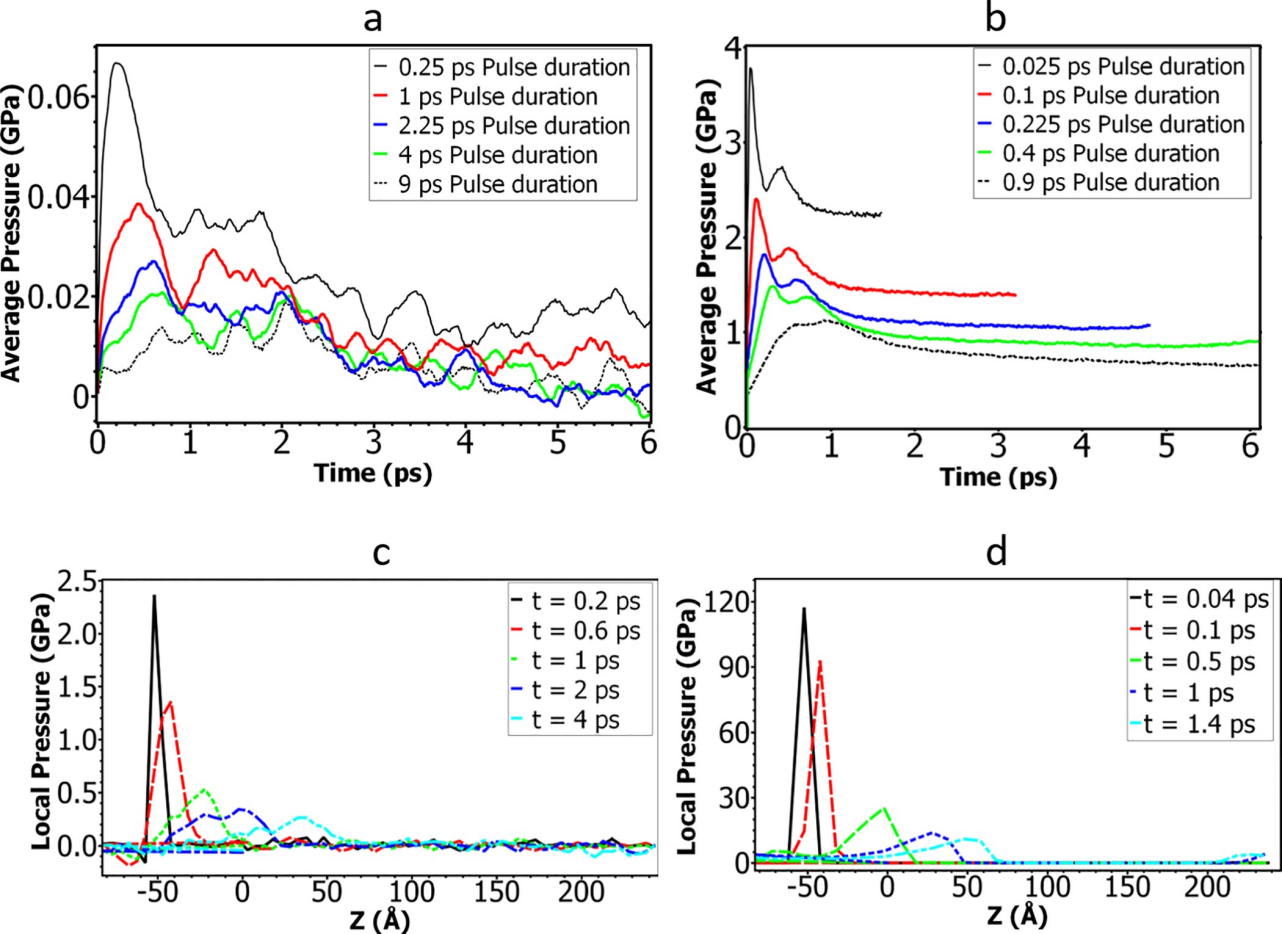
(a)-(b): Time history of the average pressure of the simulation box for different shock pulse durations when pressure impulses of (a) 1 mPa·s or (b) 10 mPa·s are applied. The differences in simulation times shown are due to the variations in shock wave speed since the shock simulations were set to stop when the wave reached the end of the simulation box. (Note that a moving average over 0.25 ps is applied to the pressure history curves.) (c)-(d): Local pressure distribution along the z direction at different times for (c) 1 mPa·s and (d) 10 mPa·s pressure impulses and for the shock water slab thickness of 5 Å.

The large kinetic energy pumped into the system with a shock impulse increases the pressure of the system. To characterize the pressure increases associated with the shock impulses, we plot the average pressure of the whole simulation box as a function of time in Fig 4A and 4B, with a moving average over 0.25 ps applied to the curves. For the same pressure impulses as seen in Fig 4A and 4B, as the pulse duration is increased, the magnitude of the peak pressure is decreased. Note that the pressure presented in Fig 4A and 4B is a mean value of the non-uniformly distributed pressure in the whole simulation box since the shock wave velocity is only added to the shock slab of a relatively small volume. The decay of the mean pressure indicates that the initially concentrated kinetic energy in the shock slab has been converted into the potential energy.

To estimate the shock wave propagation speed, we plotted the local pressure along the z direction in Fig 4C and 4D, where the 5 Å length water slab was used, and the bin size used for calculating the local pressure is 5 Å. One can see that although the shock waves are applied through a water slab with uniformly added velocities, the spatial profile of the local pressure has quickly become a triangular shape at *t* = 0.2 ps for 1 mPa·s impulse (Fig 4C) and at *t* = 0.04 ps for 10 mPa·s impulse (Fig 4D). Note that the left edge of the membrane is located at -43 Å, and the triangular pressure profile has developed before it reaches the membrane. The duration of the wave increases over time, while the peak pressure of shock waves decays as time goes on. The speeds of the shock waves estimated from these figures are 3 km/s for the 1mPa·s impulse and 8 km/s for the 10 mPa·s impulse. Note that the local pressure plotted in Fig 4C and 4D is in the same order of magnitude as that predicted by Eq] (4).

**Fig 5:**
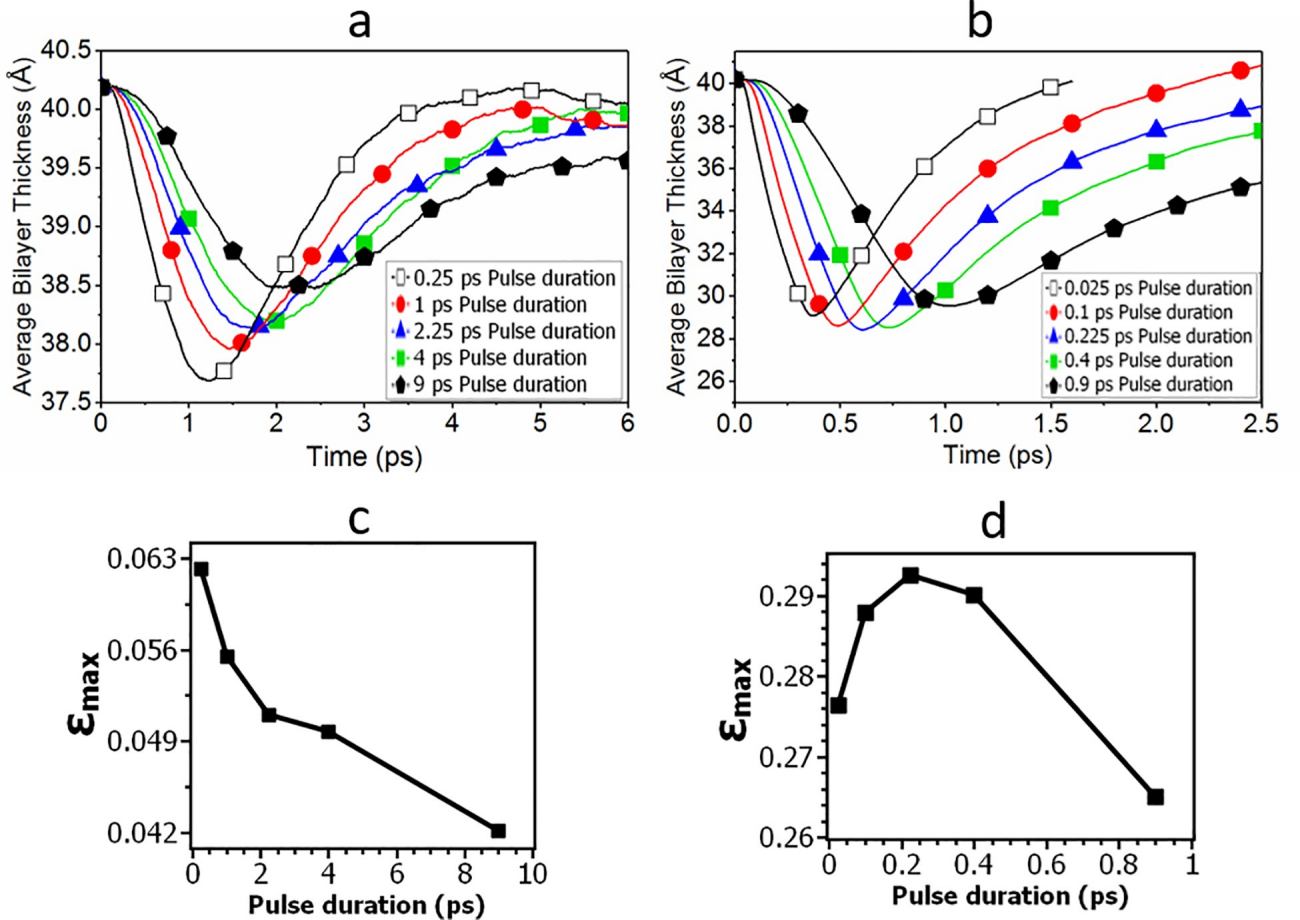
Average membrane thickness of the lipid bilayer at a pressure impulse of (a) 1 mPa·s and (b) 10 mPa·s, calculated each 0.01 ps. The maximal compressive strain at various pulse durations for the pressure impulse of (c) 1 mPa·s and (d) 10 mPa·s.

The average membrane thickness as a function of time for different pulse durations is plotted in Fig 5A and 5B, where the compression and recovery of the bilayer can be seen. We plot the maximal compressive strain (see its definition in Methods Section) as a function of pulse length in Fig 5C and 5D. The lower pressure impulse of 1 mPa·s generates a compressive strain of ~5%, and the larger pressure impulse of 10 mPa·s generates a compressive strain of ~30%. For the lower pressure impulse of 1 mPa·s (Fig 5C), the maximal compressive strain *ε*_*max*_ decreases with increasing pulse duration; in other words, it increases with increasing peak pressure (see Fig 4 for the relation between the peak pressure and pulse duration), which indicates the importance of the peak pressure. On the contrary, for the larger pressure impulse (Fig 5D), the maximal compressive strain *ε*_*max*_ first increases with pulse length, reaches a maximum at 0.225 ps pulse duration, and then decreases with increasing pulse length.

Our interpretation of results in Fig 5C and 5D is as follows. For the results in Fig 5C, the membrane deformation decreases with increasing pulse duration (i.e., decreases with the decreasing peak pressure), which can be simply explained by assuming the lipid bilayer membrane is elastic when the pressure impulse is relatively small. The deformation of elastic material is proportional to the magnitude of the external forces (ignoring the inertial forces). Therefore, in the small pressure impulse region, the membrane deformation is dominated by the peak pressure of the shock waves.

The non-monotonic behavior shown in Fig 5D indicates that for the same pressure impulse of 10 mPa·s, there can be an optimal pulse duration at which the membrane deformation is maximized. For the left half of the curve to the maximal point in Fig 5D, the membrane deformation increases with the pulse duration (i.e., increases with decreasing peak pressure). This “abnormal” response is similar to the results in Fig 4B. Our explanation is that the lipid membrane exhibits a strain-thickening response at sufficiently high strain rates (like a cornstarch-water mixture). The reciprocal of the pulse duration is the loading rate of the impact force applied on the membrane. For the relatively small pulse duration (i.e., the left half of the curve to the maximal point in Fig 5D), the loading rate is relatively high and strain rate of the membrane deformation is high. In the high strain rate region, as the loading rate increases (i.e. the pulse duration decreases), the bilayer becomes more viscous with increased strain rate and there is less time for deformation to occur in response to the shock, which leads to a smaller compressive strain. While for the right half of the curve to the maximal point in Fig 5D, the pulse duration becomes relatively large, so the loading rate becomes relatively small. As a result, the strain-thickening effect becomes ineffective and the peak pressure plays the dominant effect on the membrane deformation.

### The order parameter

The thickness of the membrane relies heavily on the length of acyl chains of phospholipids. The reduction in chain lengths caused by shock wave impact occurs due to an increase in acyl chain bend disorder. As the disorder of the chain bend (i.e. torsion angles) increases, bigger voids in the lipid membrane are created allowing more space for foreign molecules to traverse the lipid membrane and increasing membrane permeability. To measure the acyl chain bend disorder, the order parameter of carbon-hydrogen bonds in acyl chains can be calculated. The order parameter for membrane models is usually calculated as a long-time average as was done for the equilibrium trajectories. Such a calculation is not appropriate here because the changes during these shock simulations occur over several picoseconds. Instead, the instantaneous order parameter is averaged over all the lipids and C-H bonds when it is calculated at each time instance for the outer and inner monolayers.

**Fig 6:**
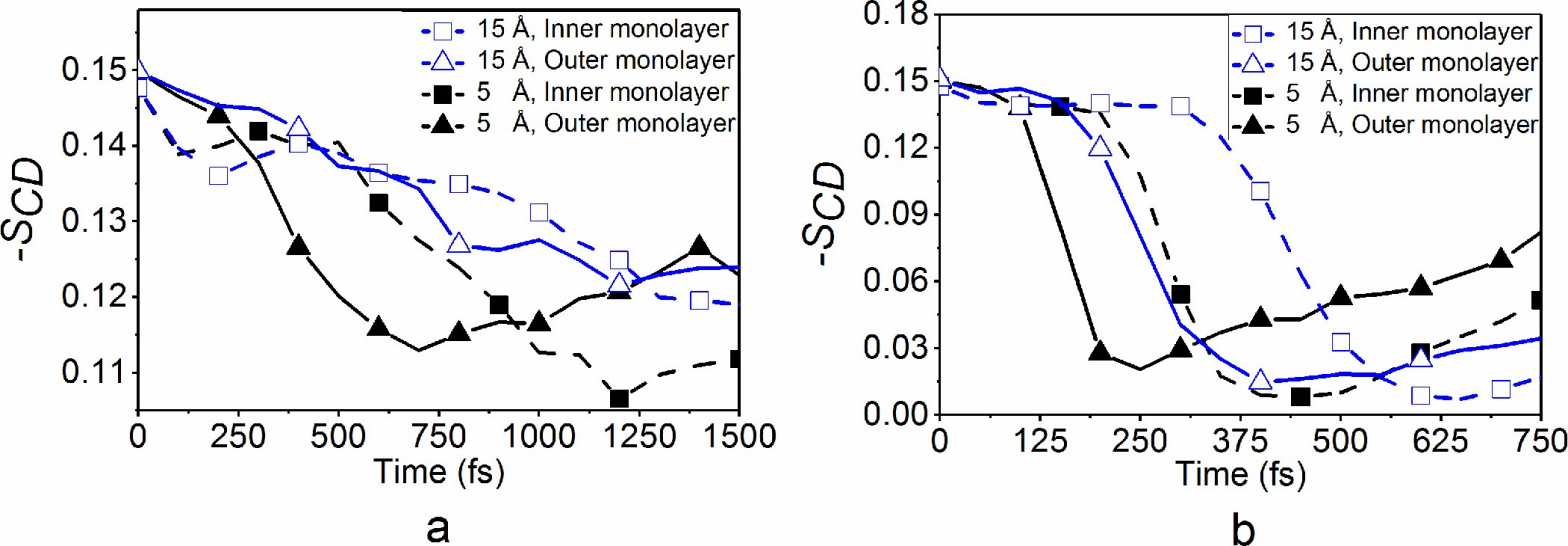
**The averaged instantaneous order parameter (-S_CD_) of the 9:1 POPC/CHL membrane model at (a) 1 mPa·s and (b) 10 mPa·s pressure impulses and shock slab thicknesses of 5 and 15 Å**.

Simulation results for the order parameter are plotted for the 9:1 POPC/CHL membrane model in Fig 6. Initially, as the shock wave impacts the lipid bilayer, the instantaneous order parameter of carbon-hydrogen bonds in acyl chains in the outer monolayer is reduced (triangle-solid lines in Fig 6). Once the shock wave momentum has propagated to the inner monolayer, the order parameters of carbon-hydrogen bonds in acyl chains in the monolayer at the right (square-dashed lines) follows with a slight time lapse in between. For the lower pressure impulse of 1 mPa·s, the larger acyl chain disorder is achieved at the smaller pulse length of 5 Å (see Fig 6A) and therefore at the larger peak pressure. In contrast, at the larger pressure impulse of 10 mPa·s, the largest acyl chain disorder is achieved at a greater pulse length of 15 Å (see Fig 5B) (although the difference is very small). These results are consistent with the membrane thickness results indicating the importance of both the peak pressure and pulse duration for increased membrane deformation.

## Conclusions

The shock simulations conducted in this research sought to understand how changing the characteristics of the applied shock impulse such as the peak pressure and the loading rate of individual pulses affect the structural properties of the lipid membrane. Previously, Kodama et al.[2] concluded that it is the pressure impulse rather than the peak pressure of the applied shock impulse that is more crucial to altering the lipid membrane structure and permeability. Our simulation results presented here demonstrate that, depending on the magnitude of the applied pressure impulse, both the peak pressure and pulse duration of the shock are important quantities to consider when increasing membrane deformation and disordering is the goal.

Imposing a shock wave with the same pressure impulse via a range of pulse durations led to differences in pressure and lipid bilayer structure, indicating that specifying the pressure impulse itself is not sufficient for determining the impacts that can be expected on membrane properties. Increasing the pressure impulse from 1 to 10 mPa·s led to qualitatively different responses on bilayer structure and lipid disorder, suggesting nonlinear effects can arise at higher pressure impulses. In summary, our simulation results suggest that the peak pressure has a dominant effect on membrane structural changes for low pressure impulses, while for the high pressure impulses, there exists an optimal pulse duration at which membrane deformation/disordering is maximized.

Our simulations were limited to the membrane deformation/disordering (i.e., thickness change and the order parameter) at different loading rate. We did not simulate poration in the membrane or actual penetration of molecules through membrane. It is possible that the membrane poration and the change of membrane permeability also depend on the loading rate of the shock waves or the impact rate of the microjets, which are more directly related to increasing the efficiency of targeted drug/gene delivery [3], [25].

## Supporting information

S1 Movie Transient response of lipid bilayer membrane to the shock wave impact. (MPG)
